# Morphological Evolution Is Accelerated among Island Mammals

**DOI:** 10.1371/journal.pbio.0040321

**Published:** 2006-09-12

**Authors:** Virginie Millien

**Affiliations:** Redpath Museum, McGill University, Montreal, Quebec, Canada; Massey University, New Zealand

## Abstract

Dramatic evolutionary changes occur in species isolated on islands, but it is not known if the rate of evolution is accelerated on islands relative to the mainland. Based on an extensive review of the literature, I used the fossil record combined with data from living species to test the hypothesis of an accelerated morphological evolution among island mammals. I demonstrate that rates of morphological evolution are significantly greater—up to a factor of 3.1—for islands than for mainland mammal populations. The tendency for faster evolution on islands holds over relatively short time scales—from a few decades up to several thousands of years—but not over larger ones—up to 12 million y. These analyses form the first empirical test of the long held supposition of accelerated evolution among island mammals. Moreover, this result shows that mammal species have the intrinsic capacity to evolve faster when confronted with a rapid change in their environment. This finding is relevant to our understanding of species' responses to isolation and destruction of natural habitats within the current context of rapid climate warming.

## Abstract 2

That morphological evolution in mammals occurs more rapidly on islands than on the mainland is a long-held assumption, but it is now confirmed using data from a range of fossil and extant species.

## Introduction

Ever since Darwin's early observations on the Galapagos finches, islands have long been recognised as “laboratories” for the study of evolution [1]. The isolation of species sometimes results in very peculiar morphologies and often drastic size changes [2–6]. Among insular mammals, there is a general tendency for small mammals to evolve toward larger size and larger species to evolve toward smaller size [2–4,6]. This tendency has come to be known as the island rule [6]. For example, the trend of increased body size (“gigantism”) has been documented in numerous rodent taxa on islands (review in [7]). On the other hand, dwarf mammoths, elephants, hippopotami, and deer are classic cases of island evolution in the field of vertebrate palaeontology [5].

The strength of the island effect on body size evolution is usually presumed to be inversely proportional to the size of the island [8] and positively related to the degree of isolation from the mainland source of the island population [2]. Various selective forces are thought to explain the size evolution in island mammals [5,8–10]. The reduced species diversity on islands results in a reorganisation of species interactions within island communities, and island species are generally subjected to reduced predation and interspecific competition. Resource limitation may also explain body size changes in some island species.

In addition, morphological changes in some island mammals have been shown to occur rapidly [11–15]. The fossil record suggests that island species adapt to their new environment rapidly following isolation, through conspicuous changes in size and morphology [5,16]. However, most of the time, it is assumed that the rate of evolution in island species is accelerated, simply because similar dramatic changes are not observed in their mainland relatives. However, not all island evolution has been rapid: many thousands of years (between 200,000 and 400,000 y) were required for Sicilian elephants, *Elephas falconeri,* to reach a height of less than 1 m and a body mass of 100 kg—less than 1% of the mass of their mainland ancestor [17]; a similar length of time may has been required for the evolution of the 1-m-high *Homo floresiensis,* isolated on the island of Flores, Indonesia [18,19]. For these two species, the absolute changes in size are substantial, but the time spans involved are relatively long. Consistent with this observation, some authors recently pointed out the ambiguity of the exact meaning of “rapid” when dealing with evolutionary rates [20,21], e.g., “rapid relative to what?” (page 2 in [20]). To date, there has been no rigorous test of the hypothesis of rapid evolution on islands. Here, I present such a test, through a comparison of evolutionary rates between island and mainland populations from a number of mammal species and over a wide range of time scales.

## Results

### Evolutionary Rate Distribution

**Figure 1:**
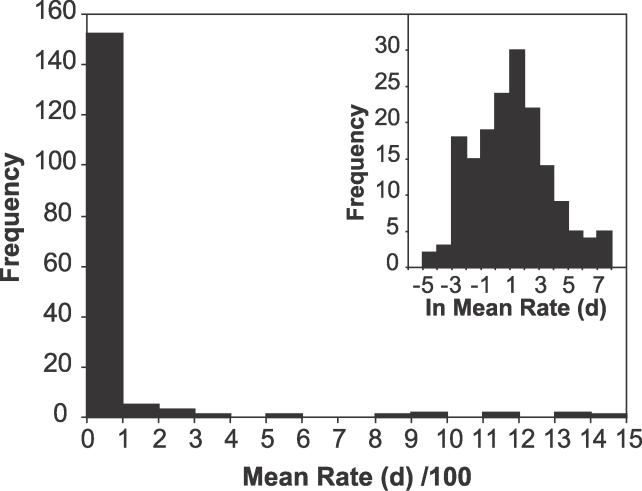
Distribution of Evolutionary Rate (darwins) Is Log-Normal for the Mean Values Dataset The distribution of evolutionary rates on a logarithmic scale is shown in the inset.

Evolutionary rates were expressed in darwins [22], and the natural logarithms of mean values of evolutionary rates were normally distributed (*n* = 170, Kolmogorov-Smirnov test, *p* = 0.9), as opposed to the distribution of the logarithms of time intervals (*p* = 0.007). The shape of the rate distribution indicated that there were many small rates and a very few large rates (Figure 1), which agrees with a previous review on contemporary microevolution (less than 300 y) [23]. The absolute values for evolutionary rates had a median of 2.88 darwins (d) and ranged from 0.014 d to 1435.85 d. This range overlaps with the range of values reported for fossil vertebrates (0.11 to 32 d) [24] and for contemporary animals (0 to 395,880 d) [25].

### Island and Mainland Rate Comparisons

**Figure 2:**
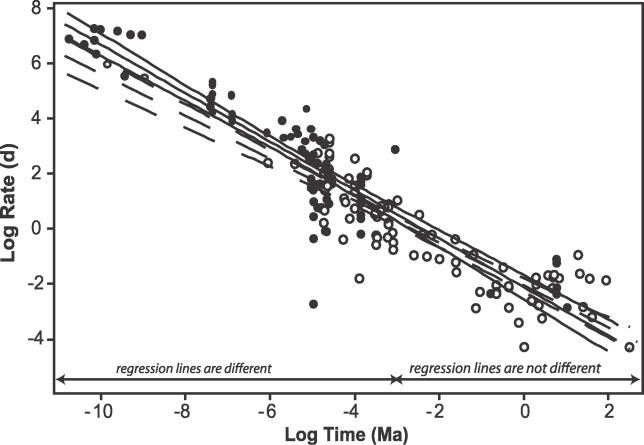
Relation between Evolutionary Rates (Mean Values, darwins) and the Time Interval (Million Years) over which They Were Calculated Filled circles: islands, open circles: mainland; the regression line for islands (solid line) is above the line for the mainland (dotted line). The 95% confidence intervals of the two regression lines do not overlap between the two groups at the smallest time intervals. The difference in elevation (i.e., the rate difference) between the two lines is statistically significant below 0.05 million years.

Significant regressions were obtained for both island and mainland species when log rate in darwins was plotted against log time interval in million years (Table 1), and evolutionary rates decreased with the time interval over which they were calculated (Figure 2). The slope of the regression of the island species was significantly less than the slope of the mainland species (Table 1). More important, the regression line of the island species was above the line of the mainland species over a large range of data (Figure 2), indicating that the evolutionary rates for island species were greater than those for mainland species. The evolutionary rate difference between the island and mainland groups were significant at the minimum, 25th percentile, and median of the time interval (Table 2). The difference between the two regression lines decreased with increasing time interval, and the difference in rates between the two groups became statistically nonsignificant for time intervals greater than 45,000 y (Table 2). As indicated on Figure 2, there are fewer island data points for the largest time intervals and fewer mainland data points for the smallest time intervals. The 95% confidence intervals of the two regression lines do not overlap over short time intervals and across the mid-range of the data. Consequently, the conclusion that island rates are faster than mainland rates is most robust over the time intervals from 21 y up to about 20,000 y.

**Table 1:**
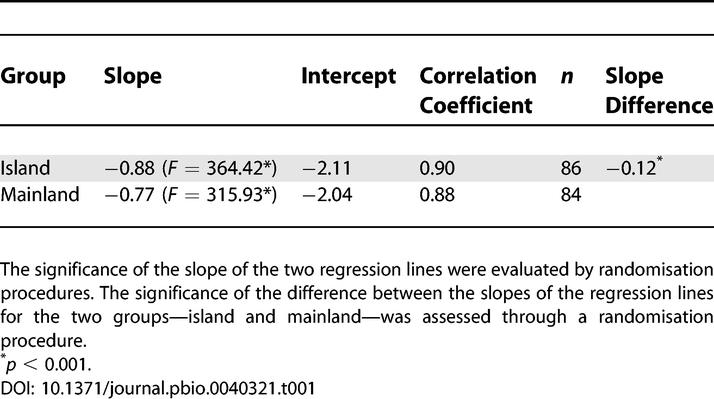
Regression Analyses of Log Evolutionary Rate (darwins) on Log Time Interval (Million Years)

**Table 2:**
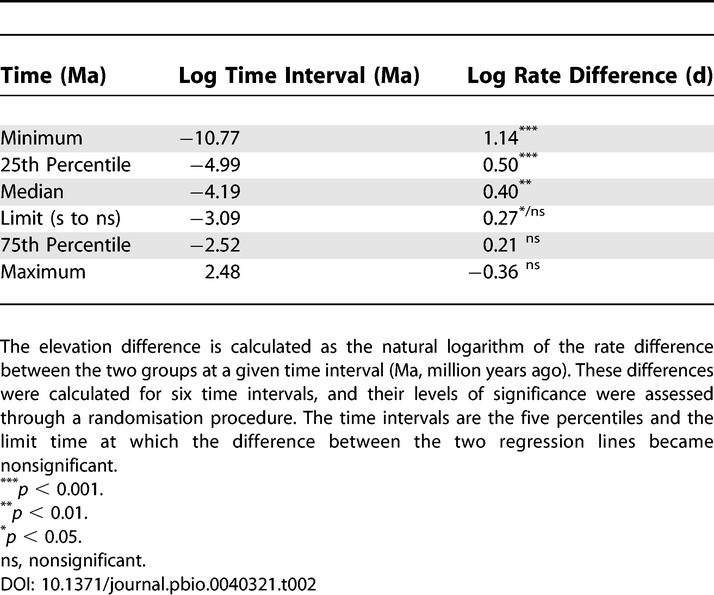
Comparisons of the Elevations of the Regression Lines for the Two Groups—Islands and Mainland—at Six Time Intervals

### The Effect of Phylogenetic Inertia

Because the data encompass a wide taxonomic range within mammals—88 species in total—one can ask whether there is a phylogenetic effect in the analyses. A test for serial independence was used to assess the phylogenetic independence of the distribution of evolutionary rate data [26]. According to this method, there was no phylogenetic autocorrelation in the dataset (Morphological tree: *C* = 0.07, *p* = 0.11; Molecular tree: *C* = 0.07, *p* = 0.10), and subsequent analyses could thus be performed on the raw dataset without correction for any phylogenetic inertia.

A closer examination of the data showed that evolutionary rate values varied within a species with locality (Protocol S1). Island evolution theory actually predicts that the effects of isolation are stronger upon populations isolated on the smallest and the most remote islands. The theory of island evolution combined with the empirical evidence on intraspecific variation presented in this review thus suggests that evolutionary rates are not strongly phylogenetically conserved, which is in accordance with the results of the test for serial independence.

### The “Rodent Effect”

Nearly 60% of the species in the dataset were rodents, and this figure is even larger when island taxa of which 74% were rodents are considered. Rodents represent nearly half of the number of living species of mammal, a fact that partially explains the bias in the dataset. A further analysis was performed to estimate the influence of the preponderance of rodents in the dataset. The evolutionary rate comparison was performed on a subsample of the dataset with equal numbers of rodent and nonrodent taxa for the two groups, island and mainland (Table 3). The slope of the regression lines of the two groups, island and mainland, were significantly different (*p* < 0.05) in 70% of the cases, and the difference between the two slopes was in most cases (75%) larger in absolute value than in the original analysis (Table 4). More important, the rate difference between the two groups, island and mainland, was always significant (*p* < 0.05) at the minimum, 25th percentile, and median of the time interval (Table 4). This last result is in accordance with the result obtained for the whole dataset. These additional analyses confirm that there is no effect of the preponderance of rodent species in the data.

**Table 3:**
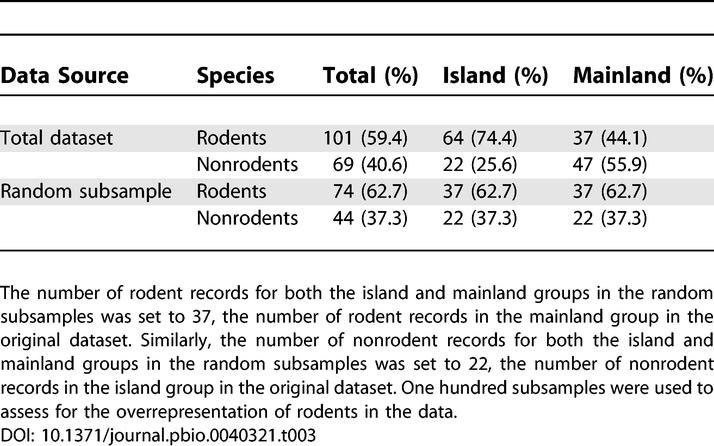
Frequencies and Percentage of Rodent and Nonrodent Records in the Total Dataset and in the Random Subsamples

**Table 4:**
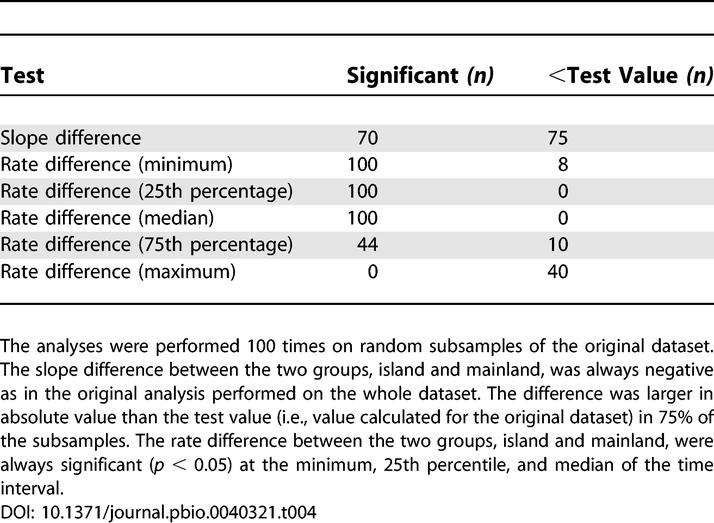
Results of the Test of the Rodent Effect

### The Influence of Geographic Variation and Dating Accuracy in the Original Data

**Figure 3:**
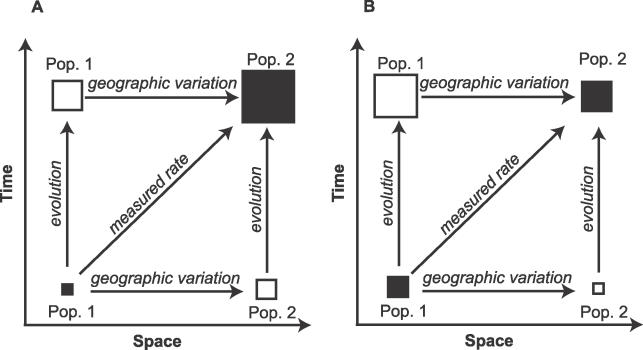
Effect of Geographic Variation on the Estimation of Evolutionary Rate (Measured Rate) when Populations from Different Localities Are Used The measured rate is the product of geographic variation (*x*-axis) across populations 1 and 2, and local evolution through time (*y*-axis) within each population. The effect of geographic variation can either increase (A) or decrease (B) the evolutionary rate estimate. The size of the square is proportional to the size of the studied character. Filled square: fossil sample preserved, open square: fossil sample not preserved.

Some large-scale studies combined data over a broad geographic range [25], in particular for some mainland species. Due to the incomplete preservation of evolutionary sequences, these studies merged data across localities, and the measured evolutionary rate is the result of the product of local evolution and geographic variation. Among mammals, body size or morphological characters often vary within a species over its distribution [27]. However, the effect of geographic variation could either increase or decrease the evolutionary rate estimate, and there is no a priori reason to expect any systematic error toward a higher or lower rate of evolution on the mainland in the dataset (Figure 3).

In addition, the time intervals were based on various methods of age estimation (see notes in Protocol S1), and it was not possible to calculate errors associated with these time intervals. However, this uncertainty did not mask the difference between mainland and island evolutionary rates, which may be an indication of the strength of the pattern of faster evolution on islands.

## Discussion

**Figure 4:**
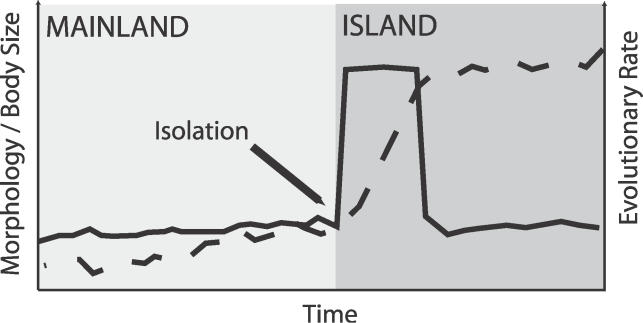
Evolution of the Size of a Morphological Character (Solid Line) in a Hypothetical Population The character size of the ancestor population from the mainland increases by a small amount on the mainland. After the isolation of the population, there is a large and rapid increase of the size of this character, and the evolutionary rate (dotted line) for this character also increases. The rate of evolution on the island then decreases to values comparable to the rate values for the mainland population.

The present data support the hypothesis that morphological evolution is accelerated among island mammals. The difference in tempo of evolution between island and mainland species also appears to be larger for shorter time intervals. These results appear to conform to the theory by which island mammals adapt to their new environment rapidly following isolation (Figure 4), through conspicuous changes in size and morphology [5,16]. Evolution seems to happen so rapidly that in most cases we do not see the intermediate between the mainland ancestor and the island endemics in the fossil record; the small likelihood of fossilisation of these intermediate forms may also be due in part to the small size of the founding population. Island species are often so distinct that we are unable to identify their specific mainland ancestor. For example, the phylogenetic relationships among dwarf elephants in the Mediterranean are still unclear [17,28]. It has been suggested that evolutionary rates among island species became smaller—even comparable to rates on the mainland—following such rapid changes [5,16,29], and my results are in accordance with this hypothesis. The commonly proposed mechanisms that govern evolution on islands—a founder event followed by slower evolution [28,29]—can be compared to the mechanisms that operate after a drastic change in the environment on the mainland [30,31]. However, the present data suggest that the peculiar ecological environment on islands—lack of predators, reduced interspecific competition, resource limitation [9,10,31–33]—favours faster evolution, even over several thousands of years. Species surviving in fragmented landscapes are also confronted with a modified environment characterised by a reduced area and an increased isolation relative to their undisturbed habitat. These new environmental conditions parallel those seen in true island habitats, and one may suspect that morphological changes in response to fragmentation are similar to changes in island species. Accordingly, body size changes in 25 Danish mammals over the last two centuries followed the island rule and were attributed to habitat fragmentation [34].

The confirmation that morphological evolution is faster on islands has some implications beyond the field of island evolution. For example, this result demonstrates that most mammal species found today on the mainland have the intrinsic capacity to evolve more rapidly. This insight suggests that the study of island species can improve our understanding of the adaptation of species to changing environmental conditions. The principal result shows that mammal species may increase their rate of morphological change by up to a factor of 3 within a few decades of dramatic and rapid change in their environment. Most species are currently confronted with an extensive deterioration and fragmentation of natural habitats. Moreover, these habitat changes are accentuated by accelerated change in the global climate [35,36]. The quantification of the rates at which species are able to evolve in response to environmental change is thus an empirical question of considerable basic and applied importance.

## Materials and Methods

### Dataset.

Data accurately documenting evolutionary rates for mammals are scarce for several reasons [37]. In particular, the fossil record rarely provides a complete sequence of evolutionary forms within a lineage of an island species. In many cases, only the final already differentiated species is known, and it cannot always be related to any fossil ancestral form. Second, given a complete evolutionary sequence, a precise dating may not be possible, which prevents the calculation of evolutionary rates. Last, even when these criteria are met, the raw data may not be available from the original publications. However, an extensive survey of the literature enabled me to calculate evolutionary rates for a number of island and mainland mammals. Experimental studies were not included in the dataset, and the calculations were based on raw data given in the text or tables or extracted from figures in 60 original publications. The dataset comprised 170 populations of 88 species or evolutionary lineages, belonging to 14 orders of mammals, and a total of 826 evolutionary rates were calculated. The characters reported in the present review included skull, skeletal, and teeth measurements, as well as external measurements. It has been established that rate values in darwins are dependent upon the dimension of the character studied [21,38]. Because rates of change for areas or volumes can not be directly compared with rates for linear measurements [38], only linear measurements have been included in the dataset. Morphometric indices, shape values such as ratios, categorical characters, or body mass were not included in the dataset. Data from males and females were distinguished in some of the original works. Data from both sexes were pooled to avoid any confounding effect of sexual dimorphism. The time intervals ranged from 21 y to 12 million y. A summary table for the entire dataset and the references for the studies used are described in Protocol S1.

### Evolutionary rates.

Evolutionary rates were expressed in darwins (d), as *(Log x2* − *Log x1)/Δt*, where a structure evolved from *x1* to *x2* over a time *Δt* in millions of years. Log is the natural logarithm [22], and the variable *x* is a linear measurement. Rates in darwins can easily be calculated from mean values published in the literature, but they are inversely related to the time interval over which they are calculated [21,24]. However, this scaling relation may be a mathematical artifact due to the plot of a ratio (rate) against its denominator *(Δt)* [39,40]. The haldane, a rate expressed in number of generations, can be used to avoid this scaling problem [24]. However, to calculate evolutionary rates in haldanes, it is necessary to know the standard deviation of the character, which is not always available in published works. The haldane also requires knowledge of generation time. For fossil species, it can be estimated from body mass through allometric relations [41], but it is questionable to study the rate of body size evolution in haldanes if the generation time is actually estimated from body size, and consequently covaries with it. Last, I used the slopes and elevations at given time intervals of the rate-time interval regressions to compare rates, which simply avoids any confounding effect of scaling on the analyses. All the rates of evolution calculated in the present review are thus in darwins. When a time series was available, the rate of evolution was estimated by the slope of the regression line of the natural logarithm of the character over *Δt*, in million years [21]. Instead of calculating many different rates for individual data points, this more conservative approach was chosen to minimise the weight of the largest studies in the analysis. Similarly, in some cases, evolutionary rate values were calculated for several characters from the same evolving population. Analyses were thus performed on the mean rate values by population and by time interval. Within a given species, only those rates initially calculated over the same time interval were pooled together to calculate mean values. There are two rationales for this approach. First, populations were considered as evolutionary independent units when isolated on different islands. Second, because evolutionary rates are related to the time interval over which they are calculated, it does not make any sense to use the average of rates that have been calculated over different time intervals. For some fossil localities, a range of time, instead of the exact datum, was given in the original work. The mean value was used for the analyses, again, to avoid statistical dependence between data points.

### Phylogenetic effect.

A test for serial independence was used to assess for the phylogenetic independence of the distribution of evolutionary rates [26]. The program PI (Phylogenetic Independence, Version 2.0) was used to conduct the test for serial independence (J. Reeve, E. Abouheif [2003] Department of Biology, McGill University, http://www.biology.mcgill.ca/faculty/abouheif). Since there is no definitive consensus on the relationships among mammals, even at the level of the order, two hypotheses—based on morphological data and based on molecular data—were considered [42]. The detailed composite phylogenies at the population level and associated bibliographic sources are given in Protocol S1. The test for serial independence was performed on the numerator data (*Log x2 – Log x1*) since evolutionary rates in darwins can not readily be used for comparisons without taking into account the denominator (*Δt*). The test for serial independence detects phylogenetic autocorrelation using the *C* statistic: *C = 1* − *(Σd^2^/Σy^2^)*, where *Σd^2^* is the sum of squared differences in rate values between successive observation on a topology and *Σy^2^* is the sum of squares [26]. For both hypotheses—morphological and molecular—the topology and associated distribution of numerator data was randomised 1,000 times and the *C* statistic was calculated for each randomised topology. The observed *C* statistic was compared to the randomised distribution to calculate its level of significance.

### Rodent effect.

There were relatively more rodent species in the data, especially in the island sample. A maximum number of 37 rodent records (which is the number of rodent records in the original mainland dataset) and 22 nonrodent records (the number of mammal other than rodents in the island dataset) were randomly selected for both island and mainland dataset. The numbers of rodent and nonrodent records were thus equal in the island and mainland samples. The rate comparison analyses were then performed on this random subsample to test for the effect of the overrepresentation of rodents in the original island dataset. The procedure was repeated 100 times, and results were compared to those obtained in the original analysis.

### Rate comparisons.

Since the distribution of the logarithms of time intervals was non-normal, all probabilities have been calculated by randomisation procedures. The regression between Log rate (darwins) and *Log time interval* (million years) was estimated for the two groups—island and mainland. Associated levels of significance of the slope were assessed by randomly reallocating the rate values to the time interval values and recalculating the slope at each iteration (1,000 iterations) [43]. The null hypothesis was a slope of zero (two-tailed test). The significance of the difference between the rate-time interval relations for the two groups was tested by randomly reallocating the (time, rate) pairs of observations between the two groups [43]. The null hypothesis was that the slopes were equal for island and mainland groups, whereas the alternative hypothesis was that the slope was larger for the island group (one-tailed test). The test statistic was the difference between the two slopes (1,000 iterations). The difference in log rate values was also calculated at five values of the time interval over the whole dataset (minimum, 25th percentile, median, 75th percentile, and maximum). These five differences were calculated for each randomised regression and compared with the five observed values in the real data. The null hypothesis was that the rates for island and mainland species were equal (two-tailed test). To determine the range of time intervals for which the difference in elevation between the two regression lines was significant, I performed a search by dichotomy to calculate the exact value of the time interval at which the difference between island and mainland evolutionary rates became nonsignificant.

## Supporting Information

Protocol S1 Table S1 Provides Dataset Used for the Analyses, and Notes Are Given on the Estimation of Time Intervals in Table S1. (463 KB DOC)
