# Correction: Morphological Evolution Is Accelerated among Island Mammals

**DOI:** 10.1371/journal.pbio.0040384

**Published:** 2006-11-14

**Authors:** Virginie Millien

In *PLoS Biology*, volume 4, issue 10: DOI: 10.1371/journal.pbio.0040321

The legend to Figure 4 incorrectly identifies the dotted line as representing evolutionary rate and the solid line as representing the size of a morphological character. This identification is reversed in the caption. The dotted line represents the morphological trait's magnitude and the solid is the rate of evolution.

