# Supplementary material for: Morphological Evolution Is Accelerated among Island Mammals
Source: PLoS Biol. 2006 Sep 12;4(10):e321. doi: 10.1371/journal.pbio.0040321 (PMC1563493; doi:10.1371/journal.pbio.0040321)
Supplement: Supplementary file 1 [file pbio.0040321.sd001.doc]

Online Supporting Information

Table S1. Data set used for the analyses. Mean values are given for evolutionary rates (darwins); N: number of rates extracted; References for sources and additional notes on the dating for each study are given below the table.

| **Taxa** | **Location** | **Time**  **(Ma)** | **N** | **Rate**  **(d)** | **NoteNote & Source** |
| --- | --- | --- | --- | --- | --- |
| **Islands** |  |  |  |  |  |
| *Apodemus argenteus* | Oki isl., Japan | 0.021 | 1 | 4.80 | 1 |
| Apodemus argenteus | Tsushima isl., Japan | 0.021 | 1 | 2.85 | 1 |
| *Apodemus argenteus* | Tane isl., Japan | 0.021 | 1 | 6.55 | 1 |
| *Apodemus argenteus* | Sado isl., Japan | 0.021 | 3 | 2.96 | 1 |
| *Apodemus argenteus* | Hokkaido isl., Japan | 0.021 | 2 | 2.54 | 1 |
| *Apodemus argenteus* | Honshu isl., Japan | 0.021 | 5 | 2.92 | 1 |
| *Apodemus gorafensis* | Gargano, Italy | 2.15 | 2 | 0.10 | 2 |
| *Apodemus speciosus* | Oki isl., Japan | 0.021 | 1 | 2.79 | 3 |
| *Apodemus speciosus* | Tsushima isl., Japan | 0.021 | 1 | 2.60 | 3 |
| *Apodemus speciosus* | Shikine isl., Japan | 0.021 | 1 | 4.88 | 3 |
| *Apodemus speciosus* | Tane isl., Japan | 0.021 | 1 | 0.83 | 3 |
| *Apodemus speciosus* | Sado isl., Japan | 0.021 | 2 | 2.73 | 3 |
| *Apodemus speciosus* | Hokkaido isl., Japan | 0.021 | 1 | 1.70 | 3 |
| *Apodemus speciosus* | Rishiri isl., Japan | 0.021 | 1 | 1.48 | 3 |
| *Apodemus speciosus* | Honshu isl., Japan | 0.021 | 4 | 5.72 | 3 |
| *Bradypus pygmaeus* | Isla Escudo, Panama | 0.0089 | 18 | 21.34 | 4 |
| *Bradypus pygmaeus* | Isla Colon, Panama | 0.0052 | 18 | 18.20 | 4 |
| *Bradypus pygmaeus* | Isla Bastimentos, Panama | 0.0047 | 18 | 31.16 | 4 |
| *Bradypus pygmaeus* | Cayo Agua, Panama | 0.0034 | 18 | 27.74 | 4 |
| *Bradypus pygmaeus* | Cayo Nancy, Panama | 0.001 | 18 | 18.06 | 4 |
| *Bradypus pygmaeus* | Isla Popa, Panama | 0.001 | 18 | 51.37 | 4 |
| *Bradypus pygmaeus* | Isla Cristobal, Panama | 0.001 | 18 | 63.92 | 4 |
| *Cervus elaphus* | Jersey | 0.0058 | 12 | 78.17 | 5 |
| *Crocidura esuae* | Sicily | 0.455 | 17 | 0.10 | 6 |
| *Herpestes javanicus* | Fiji | 0.00012 | 2 | 1135.77 | 7 |
| *Herpestes javanicus* | Okinawa | 0.00009 | 2 | 1159.07 | 7 |
| *Lepus californicus* | Carmen isl., California | 0.0093 | 1 | 5.21 | 8 |
| *Lepus californicus* | Espiritu Santo isl., California | 0.0098 | 1 | 7.80 | 8 |
| *Lepus californicus* | Magdalena isl., California | 0.0068 | 1 | 0.07 | 8 |
| *Mammuthus primigenius* | Wrangel isl. | 0.0068 | 2 | 27.96 | 9 |
| *Mammuthus primigenius* | St Paul isl. | 0.0045 | 1 | 38.1 | 10 |
| *Megaloceros giganteus* | Isle of Man, UK | 0.0023 | 2 | 33.10 | 11 |
| *Microtia* sp. | Gargano, Italy | 2.15 | 3 | 0.29 | 12/13 |
| *Microtus henseli* | Sardinia, Italy | 0.0061 | 4 | 13.28 | 14 |
| *Mus musculus* | Fugloy, Faroe isls., UK | 0.0006 | 3 | 117.2 | 15 |
| *Mus musculus* | Nolsoy, Faroe isls., UK | 0.0006 | 3 | 211.14 | 15 |
| *Mus musculus* | Mykines, Faroe isls., UK | 0.0006 | 3 | 188.53 | 15 |
| *Mus musculus* | Hestur, Faroe isls., UK | 0.0006 | 3 | 135.24 | 15 |
| *Mus musculus* | Stremoy, Faroe isls., UK | 0.0006 | 3 | 70.86 | 15 |
| *Mus musculus* | Lavezzi isl., Corsica, France | 0.0006 | 10 | 112.61 | 16 |
| *Mus musculus* | Skokholm isl., UK | 0.00007 | 3 | 1323.36 | 17 |
| *Mus musculus* | Isle of May, UK | 0.00008 | 3 | 256.70 | 17 |
| *Mus musculus* | Chichijima isl., Japan | 0.00002 | 6 | 988.67 | 18 |
| *Myomimus* sp. | Gargano, Italy | 2.15 | 2 | 0.12 | 2 |
| *Myotragus pepgonellae - M. kopperi* | Majorca, Spain | 2.8 | 4 | 0.06 | 19 |
| *Neotoma anthonyi* | Todos Santos isl., California | 0.0098 | 1 | 61.61 | 20 |
| *Neotoma bryanti* | Cedros isl., California | 0.0091 | 1 | 9.98 | 20 |
| *Neotoma bunkeri* | Coronados isl., California | 0.0064 | 1 | 37.90 | 20 |
| *Neotoma lepida* | San Jose isl., California | 0.0106 | 1 | 5.32 | 20 |
| *Neotoma lepida* | Espiritu Santo isl., California | 0.0098 | 1 | 7.73 | 20 |
| *Neotoma lepida* | Carmen isl., California | 0.0093 | 1 | 10.96 | 20 |
| *Neotoma lepida* | Danzante isl., California | 0.0080 | 1 | 24.55 | 20 |
| *Neotoma lepida* | Sta Margarita isl., California | 0.0068 | 1 | 4.85 | 20 |
| *Neotoma lepida* | Magdalena isl., California | 0.0068 | 1 | 1.56 | 20 |
| *Neotoma lepida* | San Marcos isl., California | 0.0056 | 1 | 15.63 | 20 |
| *Neotoma martinensis* | San Martin isl., California | 0.0075 | 1 | 10.81 | 20 |
| *Odocoileus virginianus* | Ossabaw isl., Georgia, USA | 0.004 | 6 | 28.51 | 21 |
| *Odocoileus virginianus* | Ste Catherine’s isl, Georgia, USA | 0.0033 | 6 | 51.01 | 21 |
| *Perognathus arenarius* | Sta Margarita isl., California | 0.0068 | 1 | 2.68 | 8 |
| *Perognathus arenarius* | Magdalena isl., California | 0.0068 | 1 | 14.78 | 8 |
| *Perognathus spinatus* | San Marcos isl., California | 0.0056 | 1 | 24.19 | 8 |
| *Perognathus spinatus* | Coronados, California | 0.0064 | 1 | 4.48 | 8 |
| *Perognathus spinatus* | Carmen, California | 0.0093 | 1 | 0.92 | 8 |
| *Perognathus spinatus* | Danzante, California | 0.008 | 1 | 5.18 | 8 |
| *Perognathus spinatus* | San Jose, California | 0.0106 | 1 | 5.13 | 8 |
| *Perognathus spinatus* | Espiritu Santo, California | 0.0098 | 1 | 6.88 | 8 |
| *Perognathus spinatus* | Magdalena, California | 0.0068 | 1 | 4.22 | 8 |
| *Peromyscus eremicus* | Coronados, California | 0.0064 | 1 | 6.15 | 8 |
| *Peromyscus eremicus* | San Jose, California | 0.0106 | 1 | 5.92 | 8 |
| *Peromyscus eremicus* | Espiritu Santo, California | 0.0098 | 1 | 2.51 | 8 |
| *Peromyscus eremicus* | Sta Margarita, California | 0.0068 | 1 | 0.72 | 8 |
| *Peromyscus eremicus* | Cedros, California | 0.0091 | 1 | 0.93 | 8 |
| *Peromyscus eva* | Carmen, California | 0.0093 | 1 | 2.94 | 8 |
| *Peromyscus maniculatus* | Sta Barbara isl., California | 0.00004 | 7 | 1381.57 | 22 |
| *Peromyscus maniculatus* | Anacapa isl., California | 0.00004 | 10 | 949.08 | 22 |
| Peromyscus maniculatus | Sta Cruz isl., California | 0.00004 | 8 | 1435.85 | 22 |
| *Peromyscus maniculatus* | Sta Margarita, California | 0.0068 | 1 | 5.04 | 8 |
| *Peromyscus maniculatus* | Magdalena, California | 0.0068 | 1 | 11.13 | 8 |
| *Peromyscus maniculatus* | San Martin isl., California | 0.0075 | 1 | 2.20 | 8 |
| *Peromyscus maniculatus* | Todos Santos isl., California | 0.00098 | 1 | 127.72 | 8 |
| *Rattus rattus* | Lavezzi isl., Corsica, France | 0.0006 | 16 | 85.60 | 16 |
| *Rattus rattus* | Sta Cruz, Galápagos | 0.00004 | 9 | 566.89 | 22 |
| *Rattus rattus* | Baltra, Galápagos | 0.00003 | 8 | 810.38 | 22 |
| *Stertomys laticrestatus* | Gargano, Italy | 2.15 | 2 | 0.33 | 2 |
| Sylvilagus bachmani | San Jose isl., California | 0.0106 | 1 | 6.09 | 8 |
| *Sylvilagus bachmani* | Cedros isl., California | 0.0091 | 1 | 9.33 | 8 |
| **Mainland** |  |  |  |  |  |
| Acdestis oweni | Santa Cruz prov., Argentina | 0.1125 | 4 | 0.81 | 23 |
| Acdestis oweni | Santa Cruz prov., Argentina | 0.195 | 2 | 0.69 | 23 |
| *Acdestis oweni* | Santa Cruz prov., Argentina | 0.09 | 6 | 0.82 | 23 |
| *Aethomys namaquensis* | Cango valley, South Africa | 0.03074 | 3 | 0.72 | 24 |
| *Amblycoptus jessiae* | Teruel Basin, Spain | 0.2 | 7 | 0.30 | 25 |
| *Apodemus flavicollis* | Israël | 0.7 | 1 | 0.12 | 26 |
| *Apodemus mystacinus* | Israël | 0.7 | 1 | 0.06 | 26 |
| *Apodemus sylvaticus* | Israël | 0.7 | 1 | 0.12 | 26 |
| *Blarina brevicauda* | Tennessee, USA | 0.00751 | 1 | 15.93 | 27 |
| *Cantius torresi – C. abditus* | Wyoming, USA | 1.77 | 2 | 0.19 | 28 |
| *Cantius torresi – C. abditus* | Wyoming, USA | 1.33 | 2 | 0.17 | 28 |
| *Chionomys gud* | Caucasus | 0.04415 | 1 | 0.88 | 29 |
| *Chionomys nivalis* | Spain, France | 0.01601 | 1 | 1.47 | 29 |
| *Chionomys nivalis* | France | 0.02049 | 1 | 0.17 | 29 |
| *Chionomys nivalis* | Germany, Austria | 0.03338 | 1 | 1.89 | 29 |
| *Chionomys nivalis* | Bulgaria | 0.04525 | 1 | 0.62 | 29 |
| *Chionomys nivalis* | Turkey | 0.01416 | 2 | 3.03 | 29 |
| *Crocidura flavescens* | Cango Valley, South Africa | 0.04034 | 5 | 2.18 | 24 |
| *Crocidura flavescens* | Cango Valley, South Africa | 0.00883 | 5 | 2.02 | 24 |
| *Crocuta crocuta* | British Isles | 0.073 | 1 | 0.39 | 30 |
| *Crusafontina endemica* | Teruel Basin, Spain | 0.1 | 9 | 0.37 | 25 |
| *Crusafontina endemica* | Teruel Basin, Spain | 0.2 | 3 | 0.21 | 25 |
| *Crusafontina endemica* | Teruel Basin, Spain | 0.3 | 14 | 0.40 | 25 |
| *Cryptomys hottentotus* | Cango Valley, South Africa | 0.04034 | 5 | 2.46 | 24 |
| *Cynomys* sp*.* | USA | 0.03 | 2 | 0.78 | 31 |
| *Cynomys* sp*.* | USA | 0.35 | 1 | 0.10 | 31 |
| *Cynomys* sp*.* | USA | 0.32 | 1 | 0.06 | 31 |
| *Cynomys* sp*.* | USA | 1.2 | 1 | 0.08 | 31 |
| *Dicrostonyx torquatus* | Bluefish cave, Yukon, Canada | 0.018 | 1 | 12.89 | 32 |
| *Elephas recki* | Buia, Eritrea | 1.95 | 18 | 0.12 | 33 |
| *Eomys antiquus - E. zitteli* | Quercy, France | 5 | 10 | 0.04 | 34 |
| *Equus ferus – E. caballus* | Alaska | 0.0242 | 1 | 7.76 | 35 |
| *Equus germanicus* | Yougoslavia | 0.01451 | 18 | 2.71 | 36 |
| *Holmesina* sp*.* | Florida, USA | 1.988 | 16 | 0.19 | 37 |
| *Holmesina* sp*.* | Florida, USA | 2 | 2 | 0.17 | 37 |
| *Holmesina* sp*.* | Florida, USA | 2.288 | 57 | 0.17 | 37 |
| *Homo* sp*.* | Western Europe | 0.0835 | 48 | 1.68 | 38 |
| *Homo* sp*.* | Western Europe | 0.009 | 48 | 4.15 | 38 |
| *Hyracotherium aemulor – H. grangeri* | Wyoming, USA | 0.61111 | 2 | 0.25 | 39 |
| *Isoodon obesulus* | Australia | 0.04 | 6 | 1.19 | 40 |
| *Lemmus sibiricus* | Bluefish cave, Yukon, Canada | 0.018 | 1 | 4.73 | 32 |
| *Lemmus trimucronatus* | Alberta, Canada | 0.0366 | 1 | 2.18 | 41 |
| *Macropus siva – M. agilis* | Australia | 0.01 | 1 | 14.54 | 42 |
| *Macropus titan – M. giganteus* | Australia | 0.01 | 1 | 26.17 | 42 |
| *Megantereon hesperus - Smilodon floridanus* | Eurasia & North America | 4.7 | 1 | 0.16 | 43 |
| Merychyus relictus – M. crabilli | Great Plains, USA | 12 | 14 | 0.01 | 44 |
| *Microtus grafi* | Bulgaria | 0.01385 | 1 | 0.69 | 45 |
| *Microtus miurus* | Bluefish cave, Yukon, Canada | 0.018 | 1 | 3.83 | 32 |
| *Microtus ochrogaster* | New Mexico | 0.02929 | 1 | 1.71 | 46 |
| *Microtus pennsylvanicus* | Virginia, USA | 0.024 | 4 | 7.18 | 47 |
| *Microtus xanthognathus* | Bluefish cave, Yukon, Canada | 0.018 | 1 | 2.13 | 32 |
| *Mustela eversmanni* | Yukon, Canada | 0.03355 | 11 | 2.26 | 48 |
| *Mustela nigripes* | Yukon, Canada | 0.03037 | 2 | 1.57 | 48 |
| *Myocricetodon eskihisarensis* | Turkey | 0.879 | 2 | 0.03 | 49 |
| *Myocricetodon eskihisarensis* | Turkey | 1.013 | 2 | 0.01 | 49 |
| *Myosorex varius* | Cango Valley, South Africa | 0.04034 | 4 | 1.28 | 24 |
| *Neotoma lepida* | Utah, USA | 0.0087 | 1 | 1.26 | 50 |
| *Odocoileus virginianus* | Georgia, S. Carolina, USA | 0.0044 | 6 | 10.66 | 21 |
| *Ondatra zibethicus* | USA | 3.75 | 1 | 0.20 | 51 |
| *Onychomys gidleyi – O. leucogaster* | Kansas, USA | 3.6 | 11 | 0.40 | 52 |
| *Oryctolagus cuniculus* | Australia | 0.0001 | 12 | 245.58 | 53 |
| *Osphranter cooperi – O. robustus* | Australia | 0.01 | 1 | 23.5 | 42 |
| *Panthera onca* | USA | 0.52 | 14 | 0.13 | 54 |
| *Panthera onca* | USA | 0.05 | 15 | 2.84 | 54 |
| *Perameles bougaiinville* | Australia | 0.04515 | 3 | 0.48 | 40 |
| *Perameles bougaiinville* | Australia | 0.04 | 2 | 0.73 | 40 |
| *Petrogale xanthopus* | Australia | 0.01 | 1 | 8.89 | 42 |
| *Progonomys minus – P. cathalai* | Turkey | 0.519 | 12 | 0.10 | 49 |
| *Sansanosmilus palmidens-Barbourofelis fricki* | Eurasia & North America | 7 | 1 | 0.15 | 43 |
| *Sarcophilus laniarius – S. harrisii* | Australia | 0.01 | 1 | 15.57 | 42 |
| *Schizogalerix anatolica* | Turkey | 1.532 | 19 | 0.04 | 49 |
| *Schizogalerix anatolica* | Turkey | 1.325 | 2 | 0.14 | 49 |
| *Schizogalerix anatolica* | Turkey | 1.398 | 4 | 0.06 | 49 |
| *Sciurus carolinensis* | Missouri, USA | 0.0095 | 14 | 4.80 | 55 |
| *Sigmodon* sp. | USA | 4 | 1 | 0.06 | 56 |
| *Sinapodemus sp. – S. ibrahimi* | Turkey | 0.519 | 4 | 0.10 | 49 |
| *Spaniomys* sp*.* | Santa Cruz prov., Argentina | 0.135 | 2 | 0.57 | 23 |
| *Spaniomys* sp*.* | Santa Cruz prov., Argentina | 0.03 | 2 | 0.34 | 23 |
| Sylvilagus floridanus | Missouri, USA | 0.0081 | 9 | 2.14 | 55 |
| *Tatera afra* | Cango Valley, South Africa | 0.0083 | 2 | 8.04 | 24 |
| *Thomomys talpoides* | Yellowstone Nat. Park, USA | 0.0024 | 2 | 10.96 | 57 |
| *Vulpes vulpes* | Israël | 0.0158 | 2 | 6.37 | 58 |
| *Vulpes vulpes* | Poland | 0.00005 | 8 | 395.46 | 59 |
| *Wallabia vishnu – W. bicolor* | Australia | 0.01 | 1 | 14 | 42 |

**Notes on the estimation of Time Intervals in Table S1**

1, 3, 54: Evolutionary rates were given by the author.

2, 12, 13: The age of the Gargano fauna is based on a consensus in the recent literature on a MN13 to MN13-MN14 age. The time interval ranges between 1.7 and 2.6 Ma, and the average value is 2.15 Ma.

4, 20: Times of isolation were given by the authors.

5. The maximum age is 5800 years BP.

6, 21, 25, 26, 36, 48: The age of the fossil localities were given by the authors.

7: *Herpestes javanicus* was introduced to Fiji in 1883, and to Okinawa in 1910.

8: The times of isolation are from Smith (1992).

9: The mainland sample was dated between 12850 and 12000 years BP and the island specimens were dated between 7250 and 4010 years BP.

10: The mainland sample was dated between 12850 and 12000 years BP (ref. 9) and the island specimens were dated at 7908 years BP.

11: Fossil specimens were dated at 11495 and 9225 years BP (Close-y-Garey and Ballaugh, respectively).

14: SUG was dated between 4900 and 4830 years BP and CDB between 14000 and 8000 years BP.

15: *Mus musculus* was introduced on the islands between 1000 and 250 years ago.

16: Fossil specimens were dated between 1400 and 1350 years ago.

17: *Mus musculus* was introduced on Skokholm island between 1881 and 1907, and on the Isle of May at maximum in 1885.

18: There were two periods of sampling: 1977 to 1980 and 1999.

19: *M. kopperi* was dated at 2.4 Ma by radiocarbon methods and, according to the authors, Majorca became isolated at 5.2 Ma. The maximum time interval is thus 2.8 Ma.

22, 51, 56: Time intervals were given by the authors.

23: Time intervals were extracted from Figure 2.

24: Minimum and Maximum ages of the fossil localities were extracted from Figure 3, Table 44, and Table 45.

27, 40, 55: Radiocarbon dates were given in the text.

28: The ages of the fossil levels were estimated with accumulation rate values of 295 (minimum) and 650 (maximum) meters per million years.

29: Minimum and maximum ages were given in Table 2.

30: 32. The time interval was estimated from the minimum and maximum values given in the text.

31: Estimates of ages were given in Table 7.4.

33: Minimum and maximum ages were extracted from Figure 5.

34: Age estimations were given in Vianey-Liaud (1991), PPP 85:15.

35: Radiocarbon dates were available in online Supplementary data.

37: Ages were given in Table 8.1.

38: The evolutionary rates were given by the author in Tables 3 and Table 4.

39: Minimum and maximum accumulation rates are from Clyde & Gingerich (1994).

41: The fossils were radiocarbon dated beween 40670 and 32500 years BP.

42: Evolutionary rates were given in Table 1 by the authors.

43: Ages were extracted from Figure 8.

44: The time interval was estimated at 12 Ma by the author.

45: Radiocarbon dates for layer 11 and 7/6a were given in the text.

46: Ages were given in Table 1.

47: The fossils were radiocarbon dated beween 29870 and 17880 years BP.

49: Ages of fossil localities were given in Table 2.3.

50: Minimum and maximum ages are from Table 1.

52: The ages of fossil localities were given in Table 8.

53: The time interval is 125 years, since the introduction of the wild rabbit in Australia.

57: Mean time intervals were estimated from Figure 2.

58: Minimum and Maximum ages of the fossil locality were given in Table I.

59: Four periods of collection were distinguished by the author.

**Figure S1.** Composite morphological tree for the populations studied. Ordinal [60] and infra-ordinal topologies [61–69] are from various sources.

**Figure S2.** Composite molecular tree for the populations studied. Ordinal topology [60] and infra-ordinal topologies [61-69] are from various sources.

References for Supporting Online Information

1. Millien V (2004) The relative effects of climate change, isolation and competition on body size evolution in the Japanese field mouse *Apodemus argenteus*. Journal of Biogeography 31: 1267-1276.

2. Millien V (unpublished)

3. Millien V, Damuth J (2004) Climate change and size evolution in an island rodent species: new perspectives on the island rule. Evolution 58: 1353-1360.

4. Anderson RP, Handley Jr CO (2002) Dwarfism in insular sloths: biogeography, selection, and evolutionary rate. Evolution 56: 1045-1058.

5. Lister AM (1989) Rapid dwarfism of red deer on Jersey in the last interglacial. Nature 342: 539-542.

6. Sara M (1995) The Sicilian (*Crocidura sicula*) and the Canary (*C. canariensis*) shrew (Mammalia, Soricidae): peripherical isolate formation and geographic variation. Boll. Zool. 62: 173-182.

7. Simberloff D, Dayan T, Jones C, Ogura G (2000) Character displacement and release in the small Indian mongoose, *Herpetes javanicus*. Ecology 81: 2086-2099.

8. Lawlor TE (1982) The evolution of body size in mammals: evidence from insular populations in Mexico. The American Naturalist 119: 54-72.

9. Vartanyan SL, Garutt VE, Sher AV (1993) Holocene dwarf mammoths from Wrangel Island in the Siberian Arctic. Nature 362: 337-340.

10. Guthrie RD (2004) Radiocarbon evidence of mid-Holocene mammoths stranded on an Alaskan Bering Sea island. Nature 429: 746-749.

11. Gonzalez S, Kitchener AC, Lister AM (2000) Survival of the Irish elk into the Holocene. Nature 405: 753-754.

12. Millien V, Jaeger J-J (2001) Size evolution of the lower incisor of *Microtia*, a genus of endemic murine rodents from the late Neogene of Gargano, southern Italy. Paleobiology 27: 379-391.

13. Freudenthal M (1976) Rodent stratigraphy of some Miocene fissure fillings in Gargano (prov. Foggia, Italy). Scripta Geologica 37: 1-20.

14. Mezzabotta C, Masini F, Torre D (1996) Evolution of the first lower molar in the endemic vole *Microtus* (*Tyrrhenicola*) *henseli* (Arvicolidae, Rodentia, Mammalia) from Pleistocene and Holocene localities of Sardinia and Corsica. Acta zool. cracov. 39: 357-372.

15. Berry RJ, Jakobson ME, Peters J (1978) The house mice of the Faroe Islands: a study in microdifferentiation. Journal of Zoology London 185: 73-92.

16. Vigne J-D, Cheylan G, Granjon L, Auffray J-C (1993) Evolution ostéométrique de *Rattus rattus* et de *Mus musculus domesticus* sur de petites îles: comparaison de populations médiévales et actuelles des îles Lavezzi (Corse) et de Corse. Mammalia 57: 85-98.

17. Berry RJ (1964) The evolution of an island population of the house mouse. Evolution 18: 468-483.

18. Takada Y, Tateishi T, Sakai E, Uematsu Y (2002) Morphological variation of mice, *Mus* *musculus* on the Ogasawara islands, and their relationship to those on the Izu islands, Japan. Mammal Study 27: 23-30.

19. Köhler M, Moyà-Solà S (2004) Reduction of brain and sense organs in the fossil insular bovid *Myotragus*. Brain Behav. Evol. 63: 125-140.

20. Smith FA (1992) Evolution of body size among woodrats from Baja California, Mexico. Functional Ecology 6: 265-273.

21. Purdue JR, Reitz EJ (1993) Decrease in body-size of white-tailed deer (*Odocoileus* *virginianus*) during the late Holocene in South Carolina and Georgia. In: Martin RA, Barnosky AD, editors. Morphological change in Quaternary mammals of North America. Cambridge: Cambridge University Press. pp. 281-298.

22. Pergams ORW, Ashley MV (1999) Rapid morphological change in island deer mice. Evolution 53: 1573-1581.

22. Pergams ORW, Ashley MV (2001) Microevolution in island rodents. Genetica 112-113: 245-256.

23. Anderson DK, Damuth J, Bown TM (1995) Rapid morphological change in Miocene marsupials and rodents associated with a volcanic catastrophe in Argentina. Journal of Vertebrate Paleontology 15: 640-649.

24. Avery DM (1982) Micromammals as palaeoenvironmental indicators and an interpretation of the Late Quaternary in the Southern Cape Province, South Africa. Ann. S. Afr. Mus. 85: 183-374.

25. van Dam JA (2004) Anourosoricini (Mammalia: Soricidae) from the Mediterranean region: a pre-Quaternary example of recurrent climate-controlled North-South range shifting. J. Paleont. 78: 741-764.

26. Tchernov E (1979) Polymorphism, size trends and Pleistocene paleoclimatic response of subgenus *Sylveamus* (Mammalia: Rodentia) in Israel. Israel Journal of Zoology 28: 131-159.

27. Klippel WE, Parmalee PW (1982) Diachronic variation in insectivores from Cheek Bend Cave and environmental change in Midsouth. Paleobiology 8: 447-458.

28. Clyde WC, Gingerich PD (1994) Rates of evolution in the dentition of early Eocene *Cantius*: comparison of size and shape. Paleobiology 20: 506-522.

29. Nadachowski A (1991) Systematics, geographic variation, and evolution of snow voles (*Chionomys*) based on dental characters. Acta Theriologica 36: 1-45.

30. Klein RG, Scott K (1989) Glacial/Interglacial size variation in fossil spotted hyenas (*Crocuta crocuta*) from Britain. Quaternary Research 32: 88-95.

31. Goodwin HT (1993) Patterns of dental variation and evolution in prairie dogs, genus *Cynomys*. In: Martin RA, Barnosky AD, editors. Morphological change in Quaternary mammals of North America. Cambridge: Cambridge University Press. pp. 107-133.

32. Morlan RE (1989) Paleoecological implications of Late Pleistocene and Holocene microtine rodents from the Bluefish Caves, northern Yukon Territory. Canadian Journal of Earth Science 26: 149-156.

33. Ferretti MP, Ficcarelli G, Libsekal Y, Tecle TM, Rook L (2003) Fossil elephants from Buia (Northern Afar depression, Eritrea) with remarks on the systematics of *Elephas recki* (Proboscidea, Elephantidae). Journal of Vertebrate Paleontology 23: 244-257.

34. Comte B, Vianey-Liaud M (1989) Eomyidae (Rodentia) de l'Oligocène d'Europe Occidentale. Palaeontographica Abt. A 209: 33-91.

35. Guthrie RD (2003) Rapid body size decline in Alaskan Pleistocene horses before extinction. Nature 426: 169-171.

36. Forsten A (1990) Dental size trend in an equid sample from the Sandalja II cave of northwestern Yugoslavia. Paläont. Z. 64: 153-160.

37. Hulbert RC, Morgan GS (1993) Quantitative and qualitative evolution in the giant armadillo *Holmesina* (Edentata: Pampatheriidae) in Florida. In: Martin RA, Barnosky AD, editors. Morphological change in Quaternary mammals of North America. Cambridge: Cambridge University Press. pp. 134-177.

38. Brace CL, Rosenberg KR, Hunt KD (1987) Gradual change in human tooth size in the Late Pleistocene and post-Pleistocene. Evolution 41: 705-720.

39. Gingerich PD (1993) Quantification and comparison of evolutionary rates. American Journal of Science 293-A: 453-478.

40. Price GJ (2005) Fossil bandicoots (Marsupialia, Peramelidae) and environmental change during the Pleistocene on the Darling Downs, southeastern Queensland, Australia. Journal of Systematic Palaeontology 2: 347-356.

41. Burns JA (2004) Late Pleistocene lemmings (*Lemmus trimucronatus* and *Discrostonyx groenlandicus*; Muridae: Rodentia) from Alberta, Canada. Journal of Mammalogy 85: 379-83.

42. Marshall LG, Corruccini RS (1978) Variability, evolutionary rates, and allometry in dwarfing lineages. Paleobiology 4: 101-119.

43. Martin LD (1984) Phyletic trends and evolutionary rates. Special Publication Carnegie Museum of Natural History 8: 526-538.

44. Bader RS (1955) Variability and evolutionary rate in the Oreodonts. Evolution 9: 119-140.

45. Montuire S, Brunet-Lecomte P (2004) Relation between climatic fluctuation and morphological variability in *Microtus* (*Terricola*) *grafi* (Arvicolinae, Rodentia) from Bacho Kiro (Bulgaria, Upper Pleistocene). Lethaia 37: 71-78.

46. Harris AH (1988) Late Pleistocene and Holocene *Microtus* (*Pitymys*) (Rodentia: Cricetidae) in New Mexico. Journal of Vertebrate Paleontology 8: 307-313.

47. Barnosky AD (1990) Evolution of dental traits since latest Pleistocene in meadow voles (*Microtus pennsylvanicus*) from Virginia. Paleobiology 16: 370-383.

48. Youngman PM (1994) Beringian ferrets: mummies, biogeography, and systematics. Journal of Mammalogy 75: 454-461.

49. Fortelius M, Kappelman J, Sen S, Bernor RL (2003) Geology and paleontology of the Miocene Sinap formation, Turkey. New-York: Columbia University Press. 409 p.

50. Lyman RL, O'Brien MJ (2005) Within-taxon morphological diversity in late-Quaternary Neotoma as a paleoenvironmental indicator, Bonneville Basin, Northwestern Utah, USA. Quaternary Research 63: 274-282.

51. Martin RA (1993) Patterns of variation and speciation in Quaternary rodents, In: Martin RA, Barnosky AD, editors. Morphological change in Quaternary mammals of North America. Cambridge: Cambridge University Press. pp. 226-280.

52. Carleton MD, Eshelman RE (1979) A synopsis of fossil grasshopper mice, genus Onychomys, and their relationships to recent species. Claude W. Hibbard Memorial Volume 7, Papers on Paleontology NO. 21, Ann Arbor, Michigan: Museum of Paleontology, The University of Michigan.

53. Williams CK, Moore RJ (1989) Phenetypic adaptation and natural selection in the wild rabbit, *Oryctolagus cuniculus*, in Australia. Journal of Animal Ecology 58: 495-507.

54. Seymour K (1993) Size change in North American Quaternary jaguars, In: Martin RA, Barnosky AD, editors. Morphological change in Quaternary mammals of North America. Cambridge: Cambridge University Press. pp. 343-372.

55. Purdue JR (1980) Clinal variation of some mammals during the Holocene in Missouri. Quaternary Research 13: 242-258.

56. Martin RA (1986) Energy, ecology, and cotton rat evolution. Paleobiology 12: 370-382.

57. Hadly EA (1997) Evolutionary and ecological response of pocket gophers (*Thomomys talpoides*) to late-Holocene climatic change. Biological Journal of the Linnean Society 60: 277-296.

58. Davis S (1977) Size variation of the fox, *Vulpes vulpes* in the palearctic region today, and in Israel during the late Quaternary. Journal of Zoology London 182: 343-351.

59. Szuma E (2003) Microevolutionary trends in the dentition of the Red fox (*Vulpes vulpes*). J. Zool. Syst. Evol. Research 41: 47-56.

60. Springer MS, Stanhope MJ, Madsen O, de Jong WW (2004) Molecules consolidate the placental mammal tree. TREE 19(8): 430-438.

61. Serizawa K, Suzuki H, Tsuchiya K (2000) A phylogenetic view on species radiation in Apodemus inferred from variation of nuclear and mitochondrial genes. Biochemical Genetics 38(1/2): 27-40.

62. Suzuki H, Sato JJ, Tsuchiya K, Luo J, Zhang Y-P et al. (2003) Molecular phylogeny of wood mice (Apodemus, Muridae) in East Asia. Biological Journal of the Linnean Society 80: 469-481.

63. Steppan SJ, Adkins RM, Anderson J (2004) Phylogeny and divergence-date estimates of rapid radiations in Muroid rodents based on multiple nuclear genes. Syst Biol 53(4): 533-553.

64. Jaarola M, Martinkova N, Gunduz I, Brunhoff C, Zima J et al. (2004) Molecular phylogeny of the speciose vole genus *Microtus* (Arvicolinae, Rodentia) inferred from mitochondrial DNA sequences. Molecular Phylogenetics and Evolution 33: 647-663.

65. Martin Y, Gerlach G, Schlotterer C, Meyer A (2000) Molecular phylogeny of European Muroid rodents based on complete cytochrome b sequences. Molecular Phylogenetics and Evolution 16(1): 37-47.

66. Marivaux L, Vianey-Liaud M, Jaeger J-J (2004) High-level phylogeny of early Tertiary rodents: dental evidence. Zoological Journal of the Linnean Society 142: 105-134.

67. Horovitz I, Sanchez-Villagra MR (2003) A morphological analysis of marsupial mammal higher-level phylogenetic relationships. Cladistics 19: 181-212.

68. Maddison DR, Schulz K-S, The Tree of Life Web Project (http://tolweb.org, 1996-2006)

69. Mc Kenna MC, Bell SK (1997) Classification of mammals above the species level. NY: Columbia Univ. Press, 631 p.
